# Supplementary material for: Application of Deep Neural Networks as a Prescreening Tool to Assign Individualized Absorption Models in Pharmacokinetic Analysis
Source: Pharmaceutics. 2021 May 26;13(6):797. doi: 10.3390/pharmaceutics13060797 (PMC8227048; doi:10.3390/pharmaceutics13060797)
Supplement: Supplementary file 1 [file pharmaceutics-13-00797-s001.zip › pharmaceutics-1202326-supplementary.pdf]

pharmaceutics

MDPI

Supplementary Materials: Application of Deep Neural Networks as a Prescreening Tool to Assign Individualized Absorption Models in Pharmacokinetic Analysis

Mutaz M. Jaber, Burhaneddin Yaman, Kyriakie Sarafoglou and Richard C. Brundage

NONMEM code

```
$PROB Hydrocortisone PK
; ; Individualized absorption model for Hydrocortisone
; ; The model has 3 absorption shapes
; ; Abs 1: First order
; ; Abs 2: Erlang distribution
; ; Abs 3: Mixed first/Erlang distribution
; ; Author: Mutaz Jaber <jaber038@umn.edu>
$INPUT C ID TIME TAD AMT DV CMTD CMT MDV MDV17 MDVD4 WT DX
FLAG
$DATA data.csv
$SUBR ADVAN13 TOL12
$EST METH=1 NOABORT PRINT=5 INTER MAX=10000
$COVR UNCOND

$MODEL NCOMP=7
$PK

TVCL = LOG(THETA(1)) + .75*LOG(WT/70)
MU_1 = TVCL
CL = EXP(MU_1 + ETA(1))

TVV = LOG(THETA(2)) + LOG(WT/70)
MU_2 = TVV
V = EXP(MU_2 + ETA(2))

TVKA = LOG(THETA(3))
IF(FLAG==3) TVKA = LOG(THETA(4))

MU_3 = TVKA
KA = EXP(MU_3 + ETA(3))

TVKTR = LOG(THETA(5))
IF(FLAG==3) TVKTR = LOG(THETA(6))

MU_4 = TVKTR
KTR = EXP(MU_4 + ETA(4))

FR = 1
IF(FLAG==3) THEN

TVF = THETA(7)
LGT = LOG(TVF/(1-TVF))
FR = EXP(LGT+ETA(7))/(1 + EXP(LGT+ETA(7)))
```

```
F1 = FR
F5 = 1 - FR
ENDIF

IF(FLAG==2) F1 = 1
IF(FLAG==1) F5 = 1

IF(DX==1) THEN
TVCORT = LOG(THETA(8)) + 0.75*LOG(WT/70)
ELSE
TVCORT = LOG(THETA(9)) + 0.75*LOG(WT/70)
ENDIF
MU_6 = TVCORT
CORT_IN = EXP(MU_6 + ETA(6))

TVIC50 = LOG(THETA(10))
MU_5 = TVIC50
IC50 = EXP(MU_5 + ETA(5))
GAM = 3
KEL = CL/V
S2 = V/100
; ; Fourier coefficient

A0 = 1.311
A1 = 0.35
A2 = 0.004036
B1 = 0.2445
B2 = -0.04736
KELA = 0.2467
PI = 3.141592653

A00 = A0*KELA
A01 = A1*KELA + (B1*2*PI/24)
A02 = A2*KELA + (B2*2*PI/12)
B01 = B1*KELA - (A1*2*PI/24)
B02 = B2*KELA - (B2*2*PI/12)
$DES
N1 = A01*COS(2*PI*T/24) + B01*SIN(2*PI*T/24)
N2 = A02*COS(2*PI*T/12) + B02*SIN(2*PI*T/12)

R_IN = A00 + N1 + N2
IF(R_IN <= 0) R_IN = 1E-5
CC = A(2)/S2
IF(CC <= 0) CC = 1E-5

EFF = CC**GAM/(CC**GAM + IC50**GAM)
INH = R_IN*(1-EFF)

DADT(1) = -KTR * A(1)
DADT(2) = KA*A(5) + KTR*A(6) - KEL * A(2)
DADT(3) = KTR * (A(1) - A(3))
DADT(4) = KTR * (A(3) - A(4))
DADT(6) = KTR * (A(4) - A(6))
DADT(5) = -KA * A(5)
DADT(7) = CORT_IN*INH - KEL*A(7)
```

```
$ERROR
IPRE = A(2)/S2 + A(7)
Y = IPRE *(1+EPS(1)) + EPS(2)
$THETA
(0, 22.6) ; CL
(0, 38.8) ; V
(0, 3.31) ; KA F
(0, 7.6) ; KA S
(0, 8.17) ; KTR E
(0, 5.2); KTR S
(0, 0.7, 1) ; F
(0, 1.15) ; CORT SW
(0, 3.26) ; CORT SV
(0, 1.55) ; IC50

$OMEGA
0.184 ; IIV CL
0.441 ; IIV V
0.237 ; IIV KA
0.05 ; IIV KTR
0.1 ; IIV IC50
0.98 ; CORT IN
0.7 ; F

$SIGMA 0.027 0.015
```

Individual Prediction

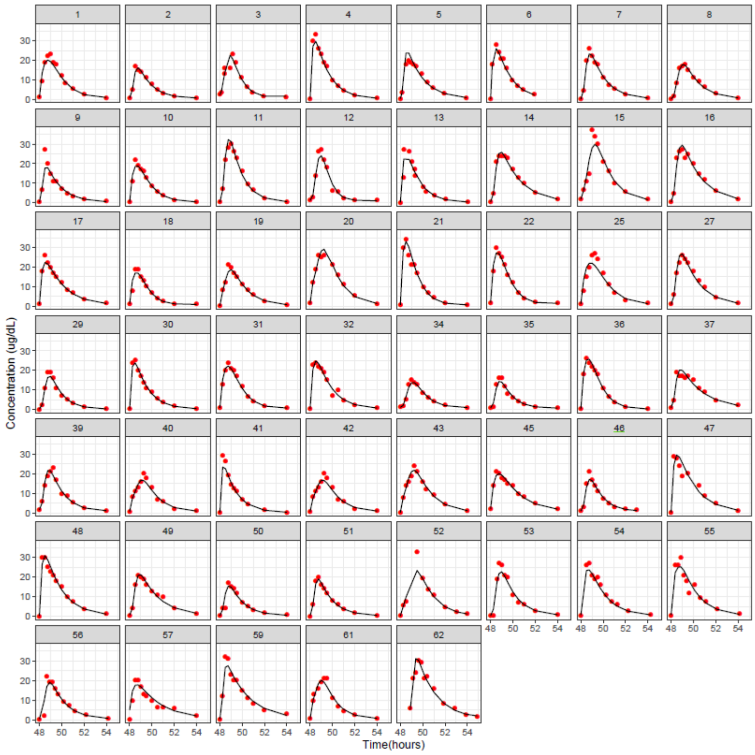

Figure S1. Individual profiles with empirical bayes estimate prediction—linear scale.

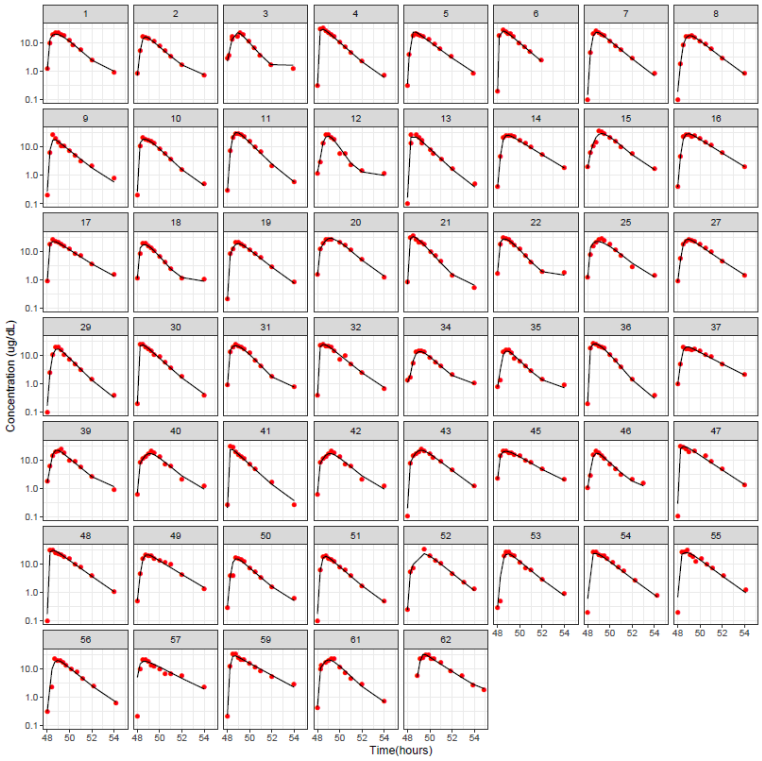

Figure S2. Individual profiles with empirical bayes estimate prediction—log scale..

R code for creating model

```
# DNN algorithm
library(tensorflow)
library(keras)
library(tidyverse)
library(splitstackshape)

# dt is the simulated data
# df is the real data

class_df <- df %>%
filter(TAD==0) %>%
select(ABSO) %>%
as_vector() %>%
unname()

df_norm <- df %>%
group_by(id) %>%
summarise(MIN=min(DV),
MAX=max(DV),
DVN = (DV-MIN)/(MAX-MIN)) %>%
ungroup()

class_df <- if_else(class_df==1, 0, if_else(class_df==2, 1, 2))

lc <- list()

for (i in 1:62) {
lc[[paste("subj",i)]] <- df_norm %>%
filter(id==i) %>%
select(DVN) %>%
as_vector() %>%
unname()
}

f <- function(data) {
nCol <- max(vapply(data, length, 0))
data <- lapply(data, function(row) c(row, rep(NA, nCol-length(row))))
data <- matrix(unlist(data), nrow=length(data), ncol=nCol, byrow=TRUE)
data.frame(data)
}

df_x$class <- class_df
df_x <- df_x[complete.cases(df_x),]
x_eval <- df_x %>% select(contains("X")) %>% as.matrix()

y_eval <- df_x %>% pull(class) %>% to_categorical(3)

x <- dt %>% select(contains("X")) %>% as.matrix()
y <- dt %>% pull(FLAG) %>% to_categorical(3)

DNN <- keras_model_sequential() %>%
layer_dense(units = 12, activation = 'relu', input=12) %>%
layer_dense(units = 64, activation = 'relu') %>%
layer_dense(units = 24, activation = 'relu') %>%
```

```
layer_dense(units = 3, activation = 'softmax')

DNN %>%
compile(
optimizer = "adam",
loss = "categorical_crossentropy",
metrics= c("accuracy"))

NN_TRAIN <- fit(DNN, x,y, validation.split=0.3, batch_size=32,epochs=100)
```
